# Public Knowledge, Attitudes, and Practices Related to COVID-19 in Iran: Questionnaire Study

**DOI:** 10.2196/21415

**Published:** 2021-02-23

**Authors:** Mohsen Abbasi-Kangevari, Ali-Asghar Kolahi, Seyyed-Hadi Ghamari, Hossein Hassanian-Moghaddam

**Affiliations:** 1 Social Determinants of Health Research Center Shahid Beheshti University of Medical Sciences Tehran Iran

**Keywords:** COVID-19, SARS-CoV-2, attitudes, coronavirus, knowledge, perceptions, practices, Iran

## Abstract

**Background:**

The COVID-19 pandemic is a rapidly growing outbreak, the future course of which is strongly determined by people’s adherence to social distancing measures.

**Objective:**

The objective of this study was to determine the knowledge level, attitudes, and practices of the Iranian population in the context of COVID-19.

**Methods:**

A nationwide study was conducted from March 24 to April 3, 2020, whereby data were collected via an online self-administered questionnaire.

**Results:**

Responses from 12,332 participants were analyzed. Participants’ mean knowledge score was 23.2 (SD 4.3) out of 30. Most participants recognized the cause of COVID-19, its routes of transmission, its symptoms and signs, predisposing factors, and prevention measures. Social media was the leading source of information. Participants recognized the dangers of the situation and felt responsible for following social distancing protocols, as well as isolating themselves upon symptom presentation. Participants’ mean practice score was 20.7 (SD 2.2) out of 24. Nearly none of the respondents went on a trip, and 92% (n=11,342) washed their hands before touching their faces.

**Conclusions:**

Knowledge of COVID-19 among people in Iran was nearly sufficient, their attitudes were mainly positive, and their practices were satisfactory. There is still room for improvement in correcting misinformation and protecting people from deception. Iranians appear to support government actions like social distancing and care for their and others’ safety.

## Introduction

The COVID-19 pandemic is a rapidly growing outbreak that has resulted in more than 13 million reported cases worldwide, as of June 2020 [1]. In response to the evolving situation, physical distancing, school closures, and lockdown measures have been implemented in many countries to contain the outbreak and prevent the overburdening of health care systems. COVID-19 has short-term and long-term socioeconomic consequences affecting the health of communities, which are not yet fully understood [2].

Since the beginning, the spread of misinformation primarily via social media became a significant concern among health authorities. Therefore, the World Health Organization launched a webpage addressing misbeliefs via sharable media [3]. There have been unsafe or stigmatizing attitudes toward COVID-19 or patients affected by it. In addition, there have been reports on dangerous or irresponsible practices among people, which could put communities at risk.

Given the high transmissibility of COVID-19, many control measures have been implemented worldwide. Nevertheless, the future course of the pandemic is strongly determined by people’s adherence to control measures [4], which highlights the essential role of proper knowledge, responsible attitudes, and safe practices [5].

Outbreak management requires people to follow social distancing measures, which occurs when people defer to health authorities due to proper knowledge, positive attitudes, and safe practices. Therefore, the educational needs of people should be addressed to provide training programs across the community, and their current knowledge level, attitudes, and practices need to be determined quickly, considering the rapid spread of COVID-19 [1]. The objective of this study was therefore to determine the level of knowledge, attitudes, and practices related to COVID-19 among the Iranian population via a rapid online survey.

## Methods

This community-based national study was approved by the Ethical Committee of Shahid Beheshti University of Medical Sciences (IR.SBMU.RETECH.REC.1399.020). Participation was anonymous and voluntary.

### Setting and Sampling

This survey was conducted from March 24 to April 3, 2020, in Iran. We collected data via an online self-administered questionnaire using Google Docs and circulated an online invitation post with a link to the questionnaire on popular social networks in Iran, including Telegram, Instagram, WhatsApp, Twitter, and LinkedIn. In the early days of the online circulation of the questionnaire, we requested medical students from all 31 provinces of Iran to send the invitation to others. Participants were Iranian adults who agreed to participate in the study and were currently living in Iran.

### Variables and the Questionnaire

Variables included sociodemographic characteristics, knowledge about COVID-19, attitudes toward COVID-19, and practices during the pandemic. Sociodemographic characteristics included participants’ age, sex, ethnicity, literacy, marital status, number of living children, smoking status, knowing someone with COVID-19, and province of residence.

The knowledge section included questions about symptoms of COVID-19, signs, predisposing factors related to severe outcomes of COVID-19, statements on general knowledge, a self-evaluation of one’s level of knowledge, information sources, and one’s need for further training. The attitudes section included statements about participants’ attitudes toward COVID-19. In response to these statements, participants chose an item using a 5-point Likert scale. The practices section included questions about self-isolation and care seeking, preventive measures, use of face masks when leaving one’s home, handwashing, leaving town, and general practices related to COVID-19.

A panel of experts evaluated the content validity of the questionnaire. An item discrimination analysis was conducted for each scale to eliminate items deemed too tricky or easy. Factor analysis was performed for factor structure. Separate test-retest analyses were conducted over 2 weeks for the three scales of the questionnaire. The test-retest correlation for the knowledge scale was 0.86. The Kuder-Richardson-20 was used to prevent an overestimation of internal consistency and resulted in a coefficient of 0.85. The test-retest correlation for the attitudes scale was 0.87, and the coefficient alpha was .89. The test-retest correlation for the practice scale was 0.91, and the coefficient alpha was .92. The pilot survey was conducted on 50 men and 50 women, who were recruited online via the convenience sampling method.

### Data Analysis

One point was awarded to each correct answer to score participants’ knowledge about COVID-19. To analyze participants’ attitudes, responses “I strongly agree” and “I agree” were combined to signify agreement; responses “I strongly disagree” and “I disagree” were combined to indicate disagreement. To analyze participants’ practices, 1 point was awarded for undertaking each correct measure or for not undertaking each wrong measure. For the key proportions using the exact binomial distribution, the 95% CI was reported. Categorical variables were analyzed via the chi-square test. For analyzing the differences between the means of two groups and or those of three or more groups, independent-sample *t* tests and one-way analysis of variance (ANOVA) were used, respectively. Statistical analyses were performed using SPSS (version 21, IBM Corp). A probability level of less than .05 was considered significant.

## Results

Responses from 12,332 participants were analyzed. The mean age was 33.2 (SD 9.9) years and ranged from 18-88 years (women: 32.9 [SD 9.5] years; men: 33.5 [SD 10.8] years). As many as 6403 (51.9%) participants reported that they knew someone with COVID-19. Other sociodemographic characteristics are presented in Table 1.

**Table 1:** Sociodemographic characteristics of participants.

Variable		Participants, n (%)
**Sex**		
	Female	7629 (61.9)
	Male	4703 (38.1)
**Marital status**		
	Never married	4502 (36.5)
	Engaged	282 (2.3)
	Married	7183 (58.2)
	Divorced	296 (2.4)
	Widowed	69 (0.6)
**Ethnicity**		
	Fars	7575 (61.4)
	Turk/Azari	2417 (19.6)
	Kurd	723 (5.8)
	Lor	696 (5.6)
	Other	921 (7.6)
**Literacy**		
	Illiterate	18 (0.1)
	Primary school	449 (3.6)
	Secondary school	518 (4.2)
	High school diploma	2195 (17.8)
	Associate degree	964 (7.8)
	Bachelor	4962 (40.3)
	Master	2262 (18.3)
	PhD and higher	945 (7.7)
	Hawzeh	19 (0.2)

### Knowledge

#### General Knowledge About COVID-19

The majority of participants identified the cause of COVID-19, its routes of transmission, and the role of self-isolation (Table 2).

The mean knowledge score was 23.2 (SD 4.3; range 0-30), out of a total of 30 (77%). The mean knowledge score of those with high school diplomas or higher degrees was significantly higher compared to those with less education. No significant differences were observed between knowledge scores and participants’ age, sex, province of residence, marital status, number of children, smoking status, knowing someone with COVID-19, having a household member with COVID-19, having the disease themselves, or having a family member passing away due to COVID-19 (Table 3).

**Table 2:** Participants’ responses to questions about general knowledge related to COVID-19.

Statement	Response		
	“It’s true,” n (%)	“It’s false,” n (%)	“I don’t know,” n (%)
COVID-19 is transmitted via respiratory droplets and infected surfaces.	12,140 (98.4)	66 (0.5)	126 (1.1)
Patients with COVID-19 and their close contacts should be quarantined >14 days.	12,035 (97.6)	61 (0.5)	236 (1.9)
COVID-19 could be transmitted from an infected travel companion.	11,991 (97.2)	167 (1.4)	176 (1.4)
COVID-19 is caused by a virus.	11,959 (97.0)	88 (0.7)	285 (2.3)
There are still no medications available for COVID-19 prevention.	11,074 (89.8)	357 (2.9)	901 (7.3)
There is currently no specific treatment available for COVID-19.	11,009 (89.3)	377 (3.1)	946 (7.6)
Handwashing with water alone is not enough for disinfection.	9916 (80.4)	2115 (17.2)	301 (2.4)
Solely using the hand dryer cannot kill the pathogen.	9093 (73.7)	2018 (16.4)	1221 (9.9)
Vaccination against pneumonia or influenza imposes no protection against COVID-19.	8956 (72.6)	720 (5.9)	2656 (21.5)
Eating garlic does not kill the pathogen.	8269 (67.1)	1812 (14.7)	2251 (18.2)
COVID-19 is not transmitted via insects.	6762 (54.8)	1584 (12.9)	3986 (32.3)
Viruses in the Coronaviridae family can cause zoonotic diseases.	6242 (50.6)	3284 (26.6)	2806 (22.8)
Regularly rinsing nostrils with saline has a protective effect against COVID-19.	5789 (46.9)	4014 (32.6)	2529 (20.5)
Mouth washing has a protective effect against COVID-19.	3705 (30.0)	5436 (44.1)	3191 (25.9)
COVID-19 always has mild symptoms.	2763 (22.4)	7332 (59.5)	2237 (18.1)
Using air humidifiers could kill the agent at home.	2543 (21.4)	7553 (61.3)	2136 (17.3)
Hand rubbing with alcohol-based solutions is more effective than handwashing with soap.	1936 (15.7)	9400 (76.2)	996 (8.1)
The cause of COVID-19 is the influenza virus.	1319 (10.7)	9449 (76.6)	1564 (12.7)
The severity of COVID-19 is always comparable to the common cold.	1265 (10.3)	10,238 (83.0)	829 (6.7)
People who recovered from COVID-19 will never get the disease again.	1129 (9.2)	8496 (68.8)	2707 (22.0)
Patients with COVID-19 cannot transmit the disease unless they have a fever.	1107 (8.9)	10,033 (81.4)	1192 (9.7)
All patients with COVID-19 require hospital admission and treatment.	885 (7.1)	10,809 (87.7)	638 (5.2)
Taking a shower with hot water can kill the agent inside the body.	858 (7.0)	10,265 (83.2)	1209 (9.8)
A person with no cough, fever, or dyspnea cannot carry the pathogen.	801 (6.5)	10,999 (89.2)	532 (4.3)
Children do not get COVID-19.	766 (6.2)	10,832 (87.8)	734 (6.0)
Going to a steam room or sauna can kill the agent inside the body.	599 (4.9)	10,357 (83.9)	1376 (11.2)
Warming the upper airways with a blow dryer can kill the agent inside the body.	446 (3.6)	11,017 (89.3)	869 (7.1)
Drinking alcoholic beverages could kill the pathogen inside the body.	209 (1.7)	11,420 (92.6)	703 (5.7)
A person who recovered from COVID-19 does not need to follow prevention protocols.	78 (0.6)	11,923 (96.7)	331 (2.7)

**Table 3:** Determinants of the COVID-19 general knowledge score among participants.

Variable		Score (SD)		95% CI
**Sex**				
	Female	23.4 (4.1)	23.3-23.5	
	Male	22.8 (4.5)	22.7-22.9	
**Age**				
	<40 years	23.2 (4.2)	23.1-23.3	
	≥40 years	23.1 (4.4)	22.9-23.3	
**Marital status**				
	Married	23.2 (4.2)	23.1-23.3	
	Never married	23.2 (4.3)	23.1-23.4	
	Divorced	23.1 (4.6)	22.6-23.6	
	Engaged	22.5 (5.0)	22.1-23.2	
	Widowed	21.5 (5.9)	19.8-22.6	
**High school diploma**				
	Yes	23.3 (4.2)	23.2-23.4	
	No	20.4 (5.4)	19.9-20.9	
**Number of children**				
	0	23.3 (4.3)	23.3-23.4	
	1	23.4 (4.1)	23.2-23.5	
	2	23.0 (4.2)	22.9-23.2	
	≥3	22.5 (4.9)	22.1-22.9	
**Smoking**				
	Yes	22.8 (4.6)	22.6-23.2	
	No	23.3 (4.2)	23.1-23.3	
	Former smoker	22.9 (4.4)	22.5-23.4	
**Knowing someone with COVID-19**				
	Yes	23.6 (4.1)	23.5-23.7	
	No	23.4 (4.4)	23.3-23.5	
**Household member with COVID-19**				
	Yes	23.4 (4.4)	23.0-23.9	
	No	23.2 (4.3)	23.1-23.3	
**Has or has had COVID-19**				
	Yes	22.8 (5.0)	22.1-23.5	
	No	23.2 (4.2)	23.2-23.3	
	Not sure	22.9 (4.6)	22.7-23.2	
**Deceased family member due to COVID-19**				
	Yes	23.5 (4.2)	23.3-23.6	
	No	23.2 (4.3)	23.1-23.3	

#### Symptoms of COVID-19

More than 90% of participants recognized the most common symptoms of COVID-19: fever, dyspnea, and cough. In addition, 98% (n=11,846) knew that worsening dyspnea could be a red flag for severe disease outcomes. However, 221 (1.8%) reported no knowledge of the general symptoms, and 280 (2.3%) stated they did not know what the symptoms of severe disease were (Table 4).

**Table 4:** Participants’ knowledge of symptoms of COVID-19 and severe course of disease.

Symptoms			Response		
			Yes, n (%)		No, n (%)
**Symptoms (n=12,111)**					
	Fever	11,567 (95.5)		544 (4.5)	
	Dyspnea	11,403 (94.2)		708 (5.8)	
	Cough	11,030 (91.1)		1081 (8.9)	
	Loss of smell or taste	8707 (71.9)		3404 (28.1)	
	Myalgia	6928 (57.2)		5183 (42.8)	
	Malaise	6678 (55.1)		5433 (44.9)	
	Sore throat	6478 (53.5)		5633 (46.5)	
	Diarrhea	4839 (40.0)		7272 (60.0)	
	Loss of appetite	4435 (36.6)		7676 (63.4)	
	Sneeze	2613 (21.6)		9498 (78.4)	
	Rhinorrhea	2590 (21.4)		9521 (78.6)	
	Loss of consciousness	2075 (17.1)		10,036 (82.9)	
	Confusion	1968 (16.2)		10,143 (83.8)	
**Signs (n=12,052)**					
	Worsening dyspnea	11,846 (98.3)		206 (1.7)	
	Worsening coughs	8564 (71.1)		3488 (28.9)	
	Fever >5 days	8252 (68.5)		3800 (31.5)	
	Loss of consciousness	2605 (21.6)		9447 (78.4)	
	Confusion	1046 (8.7)		11,006 (91.3)	
	Productive coughs	608 (5.0)		11,444 (95.0)	

#### High-Risk Settings and Predisposing Factors of Severe COVID-19

Most participants recognized that COVID-19 could be transmitted via hospitals, religious facilities, sports complexes, and public transportation. The majority considered respiratory diseases, age >60 years, and diabetes mellitus as predisposing factors for COVID-19 complications (Table 5). However, almost half (n=5253, 44.1%) did not recognize hypertension as a predisposing factor, and 411 (3.3%) reported that they did not know what the predisposing factors were.

**Table 5:** Participants’ knowledge of predisposing factors of severe COVID-19 (n=11,921).

Factors	Response	
	Yes, n (%)	No, n (%)
Respiratory disease	10,843 (91.0)	1078 (9.0)
Age >60 years	10,674 (89.5)	1247 (10.5)
Diabetes mellitus	9659 (81.0)	2262 (19.0)
Cardiovascular disease	9277 (77.8)	2644 (22.2)
Chemotherapy	9193 (77.1)	2728 (22.9)
Extensive corticosteroids use	7993 (67.0)	3928 (33.0)
Organ transplant	7896 (66.2)	4025 (33.8)
Hypertension	6668 (55.9)	5253 (44.1)
Severe obesity	3022 (25.4)	8899 (74.6)

#### Estimation of Pathogenicity, Hospitalization Rate, Case Fatality, and Reproduction Number

Participants were asked about their perception of the percentage of people exposed to the virus who may contract, be hospitalized, or pass away due to COVID-19 infection. The median estimates were 60 (IQR 30-80), 25 (IQR 17-50), and 6 (IQR 3-20), respectively. In addition, participants reported the median number of people that an individual with COVID-19 could infect to be 10 (IQR 5-30).

#### Self-Evaluation on the Level of Knowledge About COVID-19

Participants self-reported varying levels of knowledge: sufficient (n=2542, 20.6%), fair (n=8336, 67.6%), and insufficient (n=1454, 11.8%). Subsequently, when asked whether they felt the need for further training, 8892 (72.1%) responded in the affirmative. As indicated in Table 6, Telegram and audiovisual media were the leading sources of information about COVID-19 in Iran.

**Table 6:** Participants’ sources of information about COVID-19.

Source of information	Participants, n (%)
Telegram	8740 (70.9)
Audiovisual media	8631 (70.0)
Internet search	7657 (62.1)
Instagram	5762 (46.7)
News agencies	4367 (35.4)
Family and friends	4184 (33.9)
Health care professionals	4199 (34.0)
Scientific articles	3296 (26.7)
Work	1381 (11.2)
Print media	1048 (8.5)
Pamphlets and posters	1045 (8.5)
Twitter	870 (7.1)

### Attitudes

When asked about participants’ reaction to receiving a positive diagnosis for COVID-19, 11,452 (92.9%) reported self-isolation and resting at home, 10,671 (86.5%) mentioned seeing a doctor in case symptoms worsen, and 332 (2.7%) reported continuing with daily life.

In response to a question about the likelihood of COVID-19 being manufactured for bioterrorism purposes, 2971 (24.1%) reported a very high likelihood. The remaining participants answered as follows: high (n=2928, 23.7%), fair (n=3225, 26.2%), low (n=1178, 9.5%), and very low (n=2030, 16.5%). When asked about the dangers of the current situation, responses were as follows: very high (n=5817, 47.2%), high (n=5146, 41.7%), fair (n=1238, 10.1%), low (n=103, 0.8%), and very low (n=28, 0.2%). When asked whether they thought that countries would eventually defeat the disease, 11,212 (90.9%) responded in the affirmative, while 1120 (9.1%) said no.

In response to a question asking how long it would take to control the pandemic, 131 (1.1%) reported <1 month, 2762 (22.4%) reported 1-3 months, 3917 (31.8%) reported 3-6 months, 1496 (12.1%) reported 6-9 months, 1548 (12.6%) reported 9-12 months, 2141 (17.4) reported >1 year, and 337 (2.7%) had no idea.

Most respondents had positive attitudes toward COVID-19. They were on board with the government in terms of maintaining a safe physical distance, city lockdowns, and self-isolation upon symptom onset. However, participants were mostly unsure when asked how comfortable they would be when encountering someone who has recently recovered from COVID-19 (Table 7).

**Table 7:** Attitudes of participants toward COVID-19.

Statement	Respondents, n	Response	
		“I agree,” n (%)	“I disagree,” n (%)
			
Maintaining a safe physical distance is a duty for all citizens.	12,264	12,219 (99.6)	45 (0.4)
Only people at risk of the severe form of COVID-19 need to take preventive measures.	12,184	459 (3.8)	11,725 (96.2)
As an employee, I would notify my employer upon the emergence of COVID-19 symptoms.	12,118	12,070 (99.6)	48 (0.4)
I would visit a close friend or family member with COVID-19.	12,061	75 (0.6)	11,986 (99.4)
Every household member should use their towel or facial tissue to dry their hands and face.	12,056	11,933 (99.0)	123 (1.0)
As an employer, I would agree with my employees’ sick leave if they have COVID-19 symptoms.	12,019	11,959 (99.5)	60 (0.5)
Closure of businesses, schools, and universities is an opportunity to visit family and friends.	11,958	706 (5.9)	11,252 (94.1)
Relatives of a person deceased from COVID-19 should not feel ashamed.	11,902	11,673 (98.1)	229 (1.9)
All affected cities need to go under immediate lockdown.	11,573	11,215 (97.2)	322 (2.8)
Authorities need to prioritize pressing issues like road accidents or noncommunicable diseases.	11,184	442 (4.0)	10,742 (96.0)
Pet owners need to take special care of their pets’ contact with people.	11,099	10,721 (96.6)	378 (3.4)
People with a suspected travel history or close contact should report themselves to health authorities.	11,001	10,615 (96.5)	386 (3.5)
I would rather shop online instead of shopping in person.	10,339	9809 (94.9)	530 (5.1)
I would notify health authorities if my employer insisted on the illegal reopening of business.	10,140	9830 (96.9)	310 (3.1)
In a resource-scarce setting, I would make a face mask at home to cover my nose and mouth.	9705	8278 (85.3)	1427 (14.7)
Despite keeping a safe distance, I feel uncomfortable around people who have recovered from COVID-19.	9691	5589 (57.7)	4102 (42.3)
This pandemic is divine retribution due to the sins of humankind.	8699	1553 (17.9)	7146 (82.1)
The pandemic will resolve on its own when the weather gets warmer.	7527	919 (7.5)	6608 (87.8)

### Practices

#### General Practices Related to COVID-19

The mean score for general practices related to COVID-19 was 20.7 (SD 2.2, range 2-24) out of 24 (86.2%) (Table 8).

No significant differences were observed between practice scores of participants and their sex, age, province of residence, marital status, number of living children, smoking status, whether they knew someone with COVID-19, whether they had a household member with COVID-19, whether they caught the disease themselves, or whether a family member had passed away due to COVID-19 (Table 9).

**Table 8:** General statements regarding participants’ practices pertaining to COVID-19.

Statement	Response	
	Yes, n (%)	No, n (%)
I will not go on a trip during Nowruz.	11,945 (96.9)	387 (3.1)
I will isolate myself at home if I have a symptom of COVID-19.	11,878 (96.3)	454 (3.7)
I cover my mouth and nose when sneezing or coughing.	11,694 (94.8)	638 (5.2)
I disinfect the packaging of the goods I buy from stores.	11,626 (94.3)	706 (5.7)
I did not go on a trip during Nowruz.	11,468 (93.0)	864 (7.0)
I always wash my hands or use hand sanitizers before touching my face.	11,342 (92.0)	990 (8.0)
As requested by authorities, I stayed at home and isolated myself immediately.	11,208 (90.9)	1124 (9.1)
I regularly disinfect my cell phone according to guidelines.	10,412 (84.4)	1920 (15.6)
I carry hand sanitizers or water-soap solutions when outside the home.	10,357 (84.1)	1957 (15.9)
I always use a face mask if I must leave the house for an emergency.	9519 (77.2)	2813 (22.8)
I heat bread before use to kill potential pathogens.	9512 (77.1)	2820 (22.9)
I regularly disinfect highly touched surfaces at home.	9499 (77.0)	2833 (23.0)
I bought some face masks for personal protection.	8638 (70.0)	3694 (30.0)
I keep my cell phone in my pocket to reduce the chance of infection.	8251 (66.9)	4081 (33.1)
I made homemade face masks for me/my family.	2392 (19.4)	9940 (80.6)
I bought some unprescribed drugs to prevent the disease.	2065 (16.7)	10,267 (83.3)
I let my children visit their grandparents.	1573 (12.8)	10,759 (87.2)
I went to the bazaar to shop on days before Nowruz.	1176 (9.5)	11,156 (90.5)
I drank alcoholic beverages for disinfection.	301 (2.4)	12,031 (97.6)
I attended a relative’s funeral.	152 (1.2)	12,180 (98.8)
I visited a cemetary on the last Thursday before Nowruz.	138 (1.1)	12,194 (98.9)
As always, I visited my family and friends during Nowruz.	128 (1.0)	12,204 (99.0)
I let my children play with other children outside the house.	107 (0.9)	12,225 (99.1)
As always, I will visit my family and friends during Nowruz.	84 (0.7)	12,248 (99.3)

**Table 9:** Determinants of the COVID-19 general practices score among participants.

Variable		Score (SD)	95% CI
**Sex**			
	Female	20.7 (1.9)	20.5-20.8
	Male	20.4 (2.1)	20.3-20.6
**Age**			
	<40 years	20.9 (1.9)	20.8-21.1
	≥40 years	20.6 (2.2)	20.4-20.9
**Marital status**			
	Married	20.8 (2.1)	20.8-20.9
	Never married	20.5 (2.2)	20.4-20.5
	Divorced	20.8 (2.2)	20.4-20.9
	Engaged	20.3 (2.5)	20.1-20.6
	Widowed	20.8 (2.2)	20.4-20.9
**High school diploma**			
	Yes	20.6 (2.1)	20.4-20.6
	No	20.2 (2.8)	20.1-20.4
**Number of children**			
	0	20.5 (2.2)	20.4-20.7
	1	20.8 (2.1)	20.6-20.9
	2	20.9 (2.1)	20.8-20.9
	≥3	20.8 (2.2)	20.7-20.9
**Smoking**			
	Yes	20.4 (2.5)	20.3-20.7
	No	20.7 (2.1)	20.5-20.8
	Former smoker	20.6 (2.1)	20.4-20.8
**Knowing someone with COVID-19**			
	Yes	20.7 (2.1)	20.6-20.8
	No	20.7 (2.2)	20.6-20.7
**Household member with COVID-19**			
	Yes	20.7 (2.1)	20.5-20.9
	No	20.7 (2.2)	20.6-20.7
**Has or has had COVID-19**			
	Yes	20.3 (2.9)	19.9-20.7
	No	20.7 (2.1)	20.7-20.8
	Not sure	20.5 (2.4)	20.3-20.7
**Deceased family member due to COVID-19**			
	Yes	20.7 (2.2)	20.6-20.8
	No	20.7 (2.2)	20.6-20.7

#### Self-isolation, Care Seeking, and Preventive Measures

In total, 200 (1.6%) participants reported they became COVID-19 positive, 10,453 (84.8%) stated they did not get the disease, and 1679 (13.6%) reported that they had experienced some symptoms but took no measures. Among those who indicated an infection, 174 (87.0%) reported self-isolation at home, 99 (49.5%) reported visiting a doctor when symptoms became worse, and 19 (9.5%) reported carrying on with daily life.

When asked about what they did to prevent COVID-19, 11,760 (95.4%) participants reported staying at home, 11,687 (94.8%) practiced hand hygiene, 9691 (78.6%) maintained a safe physical distance, 9391 (76.2%) wore gloves, 8907 (72.2%) wore a face mask, 5458 (44.3%) took vitamin supplements, 3405 (27.6%) consumed herbal teas, 252 (2.0%) took the Imam Kazem drug, 57 (0.5%) performed wet cupping, and 48 (0.4%) took violet oil.

Among 6156 respondents, 2914 (47.3%) reported wearing surgical masks, 1989 (32.3%) cloth masks, 1566 (25.4%) N95 masks, and 772 (12.5%) homemade masks. In total, 728 (11.8%) respondents reported not wearing a mask. The mean duration of wearing each mask was 5.1 (SD 7.9) hours (median 3.0, IQR 2-5).

The mean number of times respondents performed daily handwashing with water and soap was 13.1 (SD 12.6; median 10, IQR 6-15). The mean duration of handwashing was 22.5 (SD 14.7) seconds (median 20, IQR 20-25). The mean number of times of daily rubbing hands with alcohol-based hand sanitizers among participants was 4.9 (SD 9.8; median 2, IQR 1-5). The mean duration of hand rubbing was 9.9 (SD 10.1) seconds (median 10, IQR 3-15).

#### Leaving One’s Home or Town

The mean number of times participants left their homes during the past week was 2.7 (SD 4.1; median 2, IQR 1-3). When questioned about the reason, 7303 (59.2%) reported going grocery shopping, 2103 (17.1%) went to work, 834 (6.8%) shopped for items other than groceries, 755 (6.1%) visited family and friends, 521 (4.2%) went sightseeing, 511 (4.1%) went to the hospital, and 460 (3.7%) left to take care of the elderly. As many as 2239 (18.2%) reported that they did not leave their home. Only 7.0% (n=327) of male participants stated they had not left town during the pandemic, which differed significantly from females (n=1912, 25.1%; *P*<.001).

In total, 9074 (73.6%) participants reported that they did not leave town since the official declaration of the outbreak. Among those who did leave town, the mean number of times of going outside the city was 6.5 (SD 14.7; median 2, IQR 1-4). When asked about their reason for leaving town, 1955 (60.0%) reported going to work, 906 (27.8%) visited family and friends, 338 (10.4%) went sightseeing, 24 (0.7%) visited holy shrines, and 22 (0.7%) reported other reasons.

## Discussion

### Knowledge

Participants’ COVID-19 knowledge scores were adequate. More than 90% recognized the cause of the disease, its routes of transmission, and measures for prevention. Since the beginning of the COVID-19 pandemic, the media has focused on three symptoms—fever, dyspnea, and cough. Hence, more than 90% recognized the three most common symptoms, similar to people in the United Kingdom and the United States [6]. However, infrequently discussed symptoms like gastrointestinal symptoms were less known to the public. A large majority (98%) knew that worsening dyspnea could be a sign for the severe form of the disease. However, 70% considered fever alarming. Although more than 80% considered respiratory diseases, age >60 years, and diabetes as predisposing factors, almost half did not recognize hypertension, despite imposing significant risk [7], and only 25% knew severe obesity to be a prognostic factor. Nearly all adults in the United Kingdom and the United States recognized that adults with underlying diseases were more likely to experience a fatal disease course [6]. Participants’ median estimate for the case fatality rate of COVID-19 was 6%, which was similar to results from the United Kingdom and the United States [6]. The observed case fatality rate is 4.9% in Iran, 5.8% in the United States, 14.1% in the United Kingdom, and 5.9% worldwide [1].

Regarding misbeliefs about the disease, about 15% thought that eating garlic would kill the pathogen. In comparison, 33% of Egyptians thought eating garlic was a proper preventive measure [8]. A study analyzing the trends in social media use among Iranians reported that the main misconceptions were about statistics, prevention and protection methods, treatment, dietary recommendations, and disease transmission [9]. Misbeliefs were not confined to people in Iran; 44% and 36% of the participants in the United States and the United Kingdom believed that using a hand dryer, rinsing the nose with saline, taking antibiotics, or gargling with mouthwash were effective prevention measures for COVID-19 [6]. In India, false claims about the use of herbal medicine, as well as religious and spiritual methods for prevention and treatment, became problematic and added to confusion [10].

The role of social media in delivering health information during the pandemic is significant. Telegram, the most popular social media platform in Iran, was the leading source of information among participants, followed by television and radio. When looking for a platform to deliver rapid health information to the public, social media appears to be promising [11]. There were no significant differences among participants’ knowledge score and their sex, ethnicity, or province of residence. This could suggest that the information origin of popular media sources was the same, led by national audiovisual media sources. However, there are studies that express the concern that the freedom of expression in social media could result in the dissemination of misbeliefs, misinformation, and deception [10]; this can be corrected with public health information campaigns that target these misconceptions [12]. The active and effective presence of health professionals and authorities on the internet and social media is essential, especially in the early days of a crisis when quality information might be limited on the internet [13].

### Attitudes

Participants’ attitudes toward COVID-19 were mainly positive, and the majority believed that the COVID-19 pandemic would eventually be controlled, which is similar to the results of a study from Wuhan, China [5]. Most participants recognized the dangers of the current situation. They agreed that all people need to maintain a safe physical distance and stay at home and that all affected cities should implement immediate lockdown. While 86% of people in Egypt considered COVID-19 to be dangerous [8], only 20% of participants in a study in Pakistan believed that the disease is dangerous [14]. In our study, participants felt inclined to wear face masks in public, as 85% reported that they would even make a face mask at home if they could not buy one. More than 90% of participants said they would self-isolate if they experienced symptoms. In comparison, 64% of participants in the United States reported they follow self-isolation protocols [6].

Nearly 50% of participants considered it highly likely that the COVID-19 is caused by a human-made pathogen for bioterrorism purposes, which is larger than the 27% of Egyptians who believe the virus was initially designed as a biological weapon [8].

### Practices

The mean practice score of participants was adequate. Most participants had left their home 1 to 3 times during the past week, mostly for grocery shopping. Almost 75% of participants reported that they had not left town since the beginning of the outbreak.

The early days of social distancing implementation coincided with the Persian New Year holiday, Nowruz, which lasts for at least 2 weeks. People displayed satisfactory cooperation with the government in not leaving their homes. More than 90% reported self-isolation at home immediately after being requested to do so by the government. Iranians perform holiday customs before and during Nowruz. Before Nowruz, they shop for new clothes, shoes, sweets, and nuts, and pay their respects to deceased beloved ones in cemeteries on the last Thursday before Nowruz. They also visit family and friends and take trips during this holiday. More than 90% reported that they did not go shopping on the days before Nowruz, and 99% said they did not visit a cemetery. Moreover, almost all participants stated that they did not visit their family and friends, and 97% reported that they did not go on a trip this Nowruz.

Almost all participants reported that they would not let their children play with other children outside their home. Hence, the social and mental needs of children need attention during lockdowns [15].

People also followed hygiene protocols correctly. The majority of participants covered their faces when sneezing or coughing and washed their hands before touching their faces. In comparison, only 38% of participants in a study in Pakistan avoided touching their face with contaminated hands [14]. Most participants (77%) used face masks and carried hand sanitizers when outside, which was lower than a study from Wuhan, China, where 98% of participants used face masks [5]. It is worth mentioning that although supply shortages in personal protective equipment in Iran was resolved after the early days of the pandemic, it was severely compromised at the beginning of the outbreak, which may explain the lower adherence to face mask use seen in our study. About 20% reported the use of homemade face masks, which could ease the supply demand for surgical or N95 masks that are needed at health care facilities.

While 95% of participants reported staying home and practicing hand hygiene to prevent COVID-19, less than 2% were involved in some sorts of deception. The most challenging to control, trending practices included the oral use of an herbal combination called the Imam Kazem drug and a method that involved inserting a ball of cotton wool drenched in violet oil into the anus. The use of all unapproved medications is under question due to possible serious consequences [16].

About 2% of participants reported drinking alcoholic beverages for disinfection purposes, which needs particular attention, as a new methanol poisoning outbreak is being witnessed in Iran [17]. We also noted that participation was higher among women. Gender-based differences in participation have been reported in other studies as well [18,19].

### Strengths and Limitations

This is the first nationwide study to assess the knowledge level, attitudes, and practices of people in Iran in the context of COVID-19. The strengths of this study lie in its large sample size and time of data gathering (ie, in the early days of the outbreak in Iran). Findings could empower public health authorities to precisely direct relevant information campaigns to eliminate misconceptions.

There are some limitations that must be acknowledged. Compared to the most recent national population statistics of Iran, our sample was overrepresentative of women and younger people. Since there was no representative online platform to connect researchers to people in Iran, we decided to conduct this study using the Google Forms platform. Despite the high penetration rate of the internet in Iran, some groups, especially the elderly and vulnerable groups in rural areas, might not have access to the platform. Participants were asked to read the questionnaire to their parents and grandparents to ensure higher participation of those groups; however, such voluntary measures are not guaranteed.

### Conclusions

Knowledge pertaining to COVID-19 was almost adequate among Iranians, their attitudes were mainly positive, and their practices were satisfactory. There is still room for improvement in correcting misinformation and protecting people from deception, especially in terms of consuming alcohol. Iranians appear to agree with government decisions, like the implementation of social distancing measures, and care for their safety and that of others. The government and health care authorities need to continue to maintain public trust.

## Abbreviations

ANOVA: analysis of variance
